# Immune to defeat

**DOI:** 10.7554/eLife.01599

**Published:** 2013-10-29

**Authors:** Michael L Reese

**Affiliations:** 1 **Michael L Reese** is at the Department of Pharmacology, University of Texas Southwestern Medical Center, Dallas, United Statesmichael.reese@utsouthwestern.edu

**Keywords:** *Toxoplasma gondii*, IRG proteins, coevolution, virulence, Mouse, Other

## Abstract

A dramatic example of the ‘arms race’ between hosts and pathogens has been observed in the response of mice to the parasite that causes toxoplasmosis.

**Related research article** Lilue J, Muller UB, Steinfeldt T, Howard JC. 2013. Reciprocal virulence and resistance polymorphism in the relationship between *Toxoplasma gondii* and the house mouse. *eLife* **2**:01298. doi: 10.7554/eLife.01298

**Image** Certain alleles of the protein Irgb2-b1 appear to block effector molecules secreted by the parasite that causes *Toxoplasma*. This allows other IRG proteins, including Irga6 (shown here), to attack the parasite.

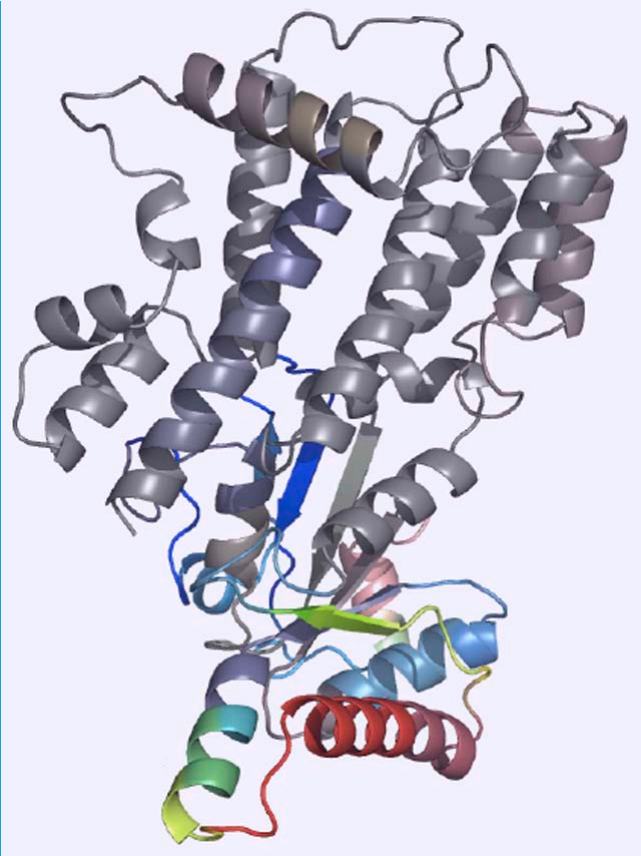

Organisms that grow in close contact with one another often co-evolve. This is particularly evident in pathogens and their hosts, with each always needing to stay one small step ahead of the other in order to survive. This evolutionary competition has given rise to the systems of immune molecules that hosts use to recognize and neutralize invading pathogens, and also to the effector molecules that pathogens use to survive attack by the immune system. A prime example of this can be seen in the interactions between a class of enzymes called the immune-related GTPases (IRG proteins) and the pathogens that they recognize and destroy (Kim et al., 2012). It is known that IRG proteins work by assembling on the surface of the vacuoles within which the pathogens replicate, but the details of this mechanism are poorly understood.

Now, in *eLife*, Jonathan Howard of the University of Cologne and colleagues present striking evidence that the co-evolution of IRG proteins with *Toxoplasma gondii*—the parasite that causes toxoplasmosis—has been an important driving force in the functional evolution of the IRG system (Lilue et al., 2013). Howard and colleagues—including Jingtao Lilue as first author, Urs Muller and Tobias Steinfeldt—found that mice derived from wild mice were resistant to strains of the parasite that were lethal to laboratory mice. Remarkably, they demonstrated that this resistance was due to genetic variation in one particular form of IRG protein, named Irgb2-b1: they found that certain alleles of Irgb2-b1 appeared to inhibit the activity of the effector molecules secreted by the parasite, which themselves have evolved to inactivate the IRG system.

As it enters a new host cell, *Toxoplasma* secretes an active kinase called ROP18 and a diverse family of pseudokinases, called ROP5. The ROP5 pseudokinases appear to sequester the IRG proteins and then present them for phosphorylation by ROP18 (Fleckenstein et al., 2012). The process of phosphorylation inactivates the IRG proteins, which prevents them from assembling on the surface of the vacuole. This, in turn, allows the parasites to replicate (Figure 1; Steinfeldt et al., 2010; Fentress et al., 2010). There are considerable differences in the virulence (to mice) of the major strains of *Toxoplasma,* and genetic variations in ROP5 and ROP18 can explain almost all of these differences (Saeij et al., 2006; Taylor et al., 2006; Behnke et al., 2011; Reese et al., 2011). This highlights the importance of the IRG system in the control of *Toxoplasma* infection in mice, and the pressure that competition with this system has placed on the parasite.

**Figure 1.**
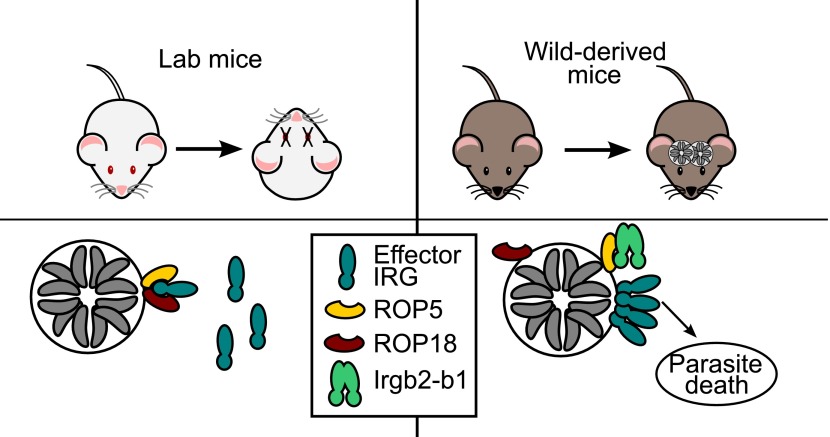
Hosts vs parasites. *Toxoplasma* parasites can kill laboratory mice (left) because they secrete effector molecules (ROP5 & ROP18) that prevent the effector IRG proteins (dark green) in the immune system of the mice from doing their job, which is to prevent the *Toxoplasma* parasites (grey) replicating themselves inside a vacuole. Lilue et al. show that in mice derived from wild mice (right), a particular isoform of an IRG protein (Irgb2-b1) binds to the ROP5 molecules, which means that the effector IRG proteins can assemble on the surface of the vacuole. This allows wild-derived mice to control an otherwise lethal infection in a way that, seemingly paradoxically, enables the survival of both the host and the parasite (which survives inside cysts in the brain and muscle tissue of the host).

As is typical of genes under strong selective pressure, the IRG genes differ both in copy number and in coding sequence in closely related species and even, as described by Lilue et al., among individuals within the same species. Previously the different forms of an IRG protein have been grouped into two distinct functional classes: effector IRG proteins that destroy the vacuoles inside which the pathogens replicate, and regulatory IRGs that are thought to inhibit premature activation of the system (Hunn et al., 2011). However, Irgb2-b1—the form of the protein identified by Lilue et al.—appears to belong to a third class of IRG proteins that recognize a particular pathogen effector rather than the pathogen itself. They found that Irgb2-b1 only attached itself to *Toxoplasma* vacuoles that were already coated with ROP5. Moreover, Irgb2-b1 loading appeared to block the activity of the ROP5 and ROP18 proteins. This left the effector IRG proteins free to assemble on the surface of the vacuole, thus leading to the destruction of the parasite (Figure 1). Strikingly, mice that carried the protective allele of Irgb2-b1 were able to survive acute infection with a highly virulent strain of the parasite, whereas infection by just one parasite of the same strain would kill a laboratory mouse.

*Toxoplasma* is also a survivor—as the acute infection ends, the parasite switches to a slow-growing form that becomes enclosed within cysts in the brain and muscle of the host, as was the case with the wild mice studied by Lilue et al. When a host is killed and eaten by a carnivore, the parasite switches back to a fast growing stage to infect its new host. Because strains of the parasite that secrete the virulent versions of both ROP5 and ROP18 are able to completely defeat the IRG system in laboratory mice, it was thought that these strains could not have co-evolved with mice, because the death of the host can be an evolutionary dead-end for a pathogen. The fact that Irgb2-b1 derived from wild mice represents a specific countermeasure to the parasite effectors provides a strong argument for co-evolution. The work of Lilue, Muller, Steinfeldt and Howard thus reminds us to look beyond lab-adapted organisms and examine biology in the wild to discover its full diversity.
